# Effects of Glucomannan on the Sacculus Rotundus and Peripheral Blood Lymphocytes in New Zealand Rabbits during Aflatoxicosis

**DOI:** 10.1100/2012/632945

**Published:** 2012-05-03

**Authors:** Emrah Sur, Hasan Hüseyin Dönmez, Murat Boydak, Mehmet Bozkurt Ataman

**Affiliations:** ^1^Department of Histology, Selçuk University Veterinary Faculty, 42031 Konya, Turkey; ^2^Department of Histology, Faculty of Veterinary Medicine, University of Kyrgyzstan-Turkey Manas, Bishkek, Kyrgyzstan; ^3^Department of Reproduction and Artificial Insemination, Selçuk University Veterinary Faculty, 42031 Konya, Turkey

## Abstract

This study was aimed to determine the effects of the glucomannan added to aflatoxin- (AF-) contaminated diet on the sacculus rotundus and peripheral blood lymphocytes of New Zealand rabbits by histological and enzyme histochemical methods. Twenty-four adult rabbits of both sexes were divided into four equal groups, namely, as control, glucomannan 0.2 g/day, AF 125 **μ**g/kg/day, and glucomannan combined with AF. The animals in all groups were treated for 12 weeks by the above-mentioned diet. When compared to control, AF-treatment caused significant decrease in alpha-naphthyl acetate esterase- (ANAE-) positive peripheral blood lymphocyte (PBL) percentages. The addition of the glucomannan to AFcontaining diet recovered the adverse effects of AF on sacculus rotundus and increased the ANAE-positive PBL counts. These results suggested that glucomannan was effective against the negative effects of AF in rabbits.

## 1. Introduction

Aflatoxins (AF), potent mycotoxins, are toxic metabolites produced by certain species of moulds, particularly *Aspergillus flavus* and *Aspergillus parasiticus*. The major source of exposure to AF is via the ingestion of contaminated food [1]. AF has carcinogenic, embryotoxic, and growth inhibitory effects and has also immunotoxic effects which cause severe economic losses in the poultry and livestock industries [2]. Immunotoxic concentration of AF is relatively lower than the levels causing retardation in growth rate [3]. So, AF contamination of the food and foodstuffs has great importance in livestock [4]. Removing AF from contaminated food and foodstuffs remains a major problem, and there is a great demand for effective decontamination technology [1, 5]. In the last decade several studies have been performed using adsorbents for detoxifying AF in contaminated food and foodstuffs [4, 6]. 

Rabbits, farming have been taken up in the world for meat, fur, and biomedical purposes, are considered quite sensitive to aflatoxicosis [7, 8]. However, there is little knowledge about changes in alpha-naphthyl acetate esterase (ANAE), a lymphocyte lysosomal enzyme [9], which has been demonstrated in mature and immunocompetent T-lymphocytes activity in peripheral blood lymphocytes and lymphoid organs, such as spleen and sacculus rotundus, enlarged terminal portion of ileum in rabbits, known as ampulla ilei or ileocecal tonsil [10, 11], and there is no studies on the ameliorative effects of dietary glucomannan, the cell-wall component of the *Saccharomyces cerevisiae*, together with aflatoxin in rabbits.

The aim of the present study was to evaluate the ameliorative effects of the glucomannan added to AF-contaminated diet on the sacculus rotundus and peripheral blood lymphocytes (PBL) of New Zealand rabbits by histological and enzyme histochemical methods, respectively.

## 2. Materials and Methods

### 2.1. New Zealand Rabbits and Diets

Twenty-four New Zealand white rabbits of both sexes, aged 12-month-old were procured from the Department of Reproduction and Artificial Insemination, Selçuk University, Konya, Turkey, and were individually housed in stainless steel cages on daylight cycle. These rabbits were fed with a toxin-free commercial base diet and water administered *ad libitum. *The rabbits were divided into four equal groups each group consisted of six animals. The rabbits received human care according to the criteria outlined in the “Guide for the Care and Use of Laboratory Animals” prepared by the National Academy of Sciences and published by the National Institute of Health. The basal diet was also tested for possible residual AF before feeding [12], and there were no detectable levels (detection limit 1 *μ*g/kg feed, recovery of the extraction method 95%).

### 2.2. Experimental Design

The experimental design consisted of four dietary treatments as follows: (1) Control (cont): Basal diet; (2) AF: basal diet plus 125 *μ*g/kg/day total aflatoxin (AF: composition given below); (3) EG: basal diet plus 0.2 g/day esterified glucomannan (Mycosorb, Alltech, KY, USA); (4) AF + EG: 125 *μ*g/kg/day aflatoxin plus 0.2 g/day esterified glucomannan. After consumption of the daily experimental diet, the animals were fed with the basal diet *ad libitum*. The groups were fed for a period of 12 weeks.

### 2.3. Aflatoxin

The AF was produced from *Aspergillus parasiticus *NRRL 2999 culture (USDA, Agricultural Research Service, Peoria, IL) via fermentation of rice by the method of Shotwell et al. [13] with minor modifications by Oğuz and Kurtoğlu [14].

### 2.4. Histological Examinations

At the end of a 12-week study, all rabbits in each group were sacrificed by cervical dislocation. Blood and lymphoid tissue samples from the *Sacculus rotundus* were taken and processed for histochemical demonstration of ANAE and for routine histological techniques, respectively.

For histological examinations, the organs were totally removed and then were trimmed midsagittally. The tissue samples were fixed in 10% buffered formaldehyde-saline solution (pH 7.4), dehydrated, and embedded in paraffin blocks. The tissue sections taken from paraffin blocks in 6 *μ*m thick were stained with Crossman's trichrome staining [15].

### 2.5. ANAE Histochemistry in the Blood Smears

ANAE was demonstrated on blood smears which were fixed in glutaraldehyde-acetone solution at −10°C for 3 minutes, according to the methods described by Maiti et al. [16]. The incubation solution was prepared by mixing 80 mL of 0.067 M phosphate buffer (pH 5.0), 4.8 mL of hexazotized pararosaniline (2.4 mL of pararosaniline (sigma) plus 2.4 mL of 4% sodium nitrite (Merck) in distilled water), and 20 mg of alpha naphthyl acetate (Sigma) in 0.8 mL of acetone. Final pH of the incubation solution was adjusted to 5.8 with 1 N NaOH.

In blood smears, the cells with lymphocyte morphology and, that have 1–3 large, reddish-brown granules were classified as ANAE-positive lymphocytes. In each blood smears 200 lymphocytes were counted. The positivity rates were expressed as percentages of the lymphocytes counted.

### 2.6. Statistical Analyses

Statistical analyses were performed with a standard computer program [17]. In order to obtain normal distribution, arc sin transformation was applied to data [18]. Differences between the arc sin transformed ANAE-positivity values of PBL were analyzed by one-way ANOVA, and importance of the differences among the groups has been determined by Duncan's multiple range test [17].

## 3. Results and Discussion

**Figure 1:**
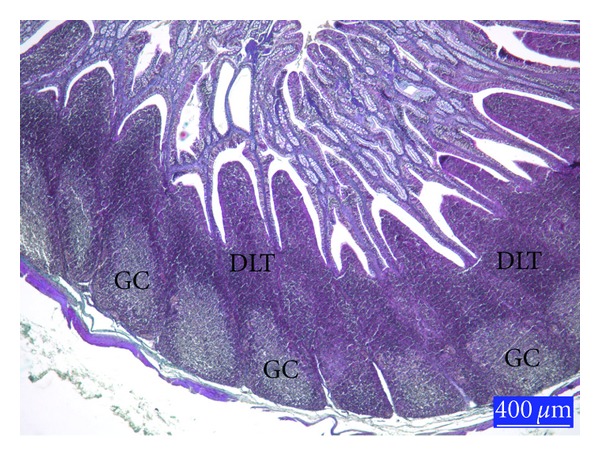
Section from the sacculus rotundus (SR) of rabbits from control group, GC: germinal center, DLT: diffuse lymphoid tissue, trichrome staining.

**Figure 2:**
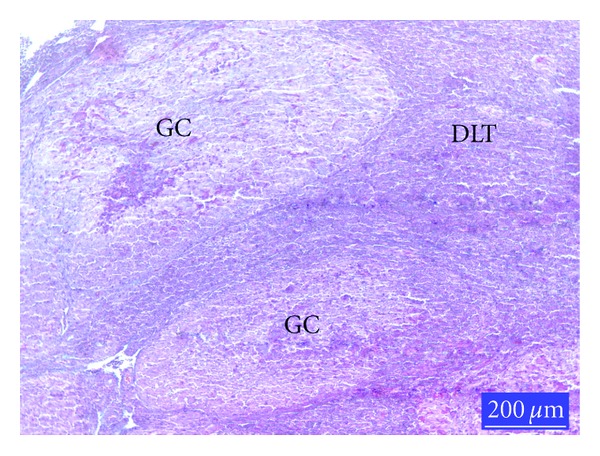
Section from the SR of rabbits from AF-treated group, GC: germinal center, DLT: diffuse lymphoid tissue; lymphoid cell depletion in GC is clear, trichrome staining.

**Figure 3:**
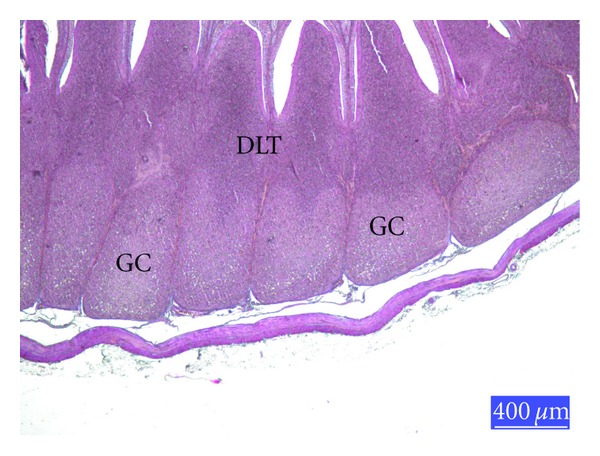
Section from the SR of rabbits from AF-plus-glucomannan-treated group, GC: germinal center, DLT: diffuse lymphoid tissue, trichrome staining.

When compared with controls (Figure 1), the lymphoid cell depletion was distinct the germinal centres (GCs) of the lymphoid follicles in the Sacculus rotundus in AF-treated group (Figure 2). Histology of the lymphoid tissues in the animals given glucomannan alone or in combination with AF was similar to the controls (Figure 3).

Immunotoxic effects of AF have been well documented in poultry [2, 4, 19]. Dietary AF induces immunosuppression in broilers and affects the thymus, bursa of Fabricius, and spleen [4, 19]. Şehu et al. [20] have reported that AF caused decrease in weight of bursa of Fabricius and spleen in quails consuming diets containing 2.5 mg/kg AF. Moreover, AF fed with diet carry over from food to eggs and other edible tissues. This situation is a threat for human health [21]. On the other hand, AF carried over from food to eggs has suppressed embryonic development of the lymphoid organs [2]. Therefore, significant functional deficiencies have been observed in the cell-mediated immune response of chicken exposed to AF during embryonic development [22].

There is a little information about the effects of AF in rabbits. However, limited studies have demonstrated that AF have serious problem in rabbit farms [8]. Krishna et al. [7] have reported an outbreak of aflatoxicosis in Angora rabbits. In this outbreak, it was observed that the level of AFB_1_ in feed samples from various farms varied from 90 to 540 *μ*g/kg feed. This outbreak of aflatoxicosis resulted of heavy morbidity and mortality which was more in weaners than in adults.

ANAE-positive peripheral blood lymphocyte levels decreased significantly (*P* < 0.05) in animals fed with AF-containing diet. Glucomannan alone had no effects on ANAE-positive lymphocyte levels although the mean values were slightly lower than controls. The percentages of ANAE-positive lymphocytes in glucomannan plus AF group were significantly higher than those of the AF group (*P* < 0.05). However, the rabbits fed with glucomannan combination with AF showed lower (*P* < 0.05) mean ANAE-positive lymphocyte levels than that of controls (Table 1).

Immunotoxic dose levels of AF are relatively lower than the levels causing retardation in growth rate. AF contaminations have emerged as one of the important factors responsible for threatening livestock production programmes. The serious economic loss caused by lowered productivity and increased mortality has become the most important problem for the farmers [3]. Therefore, AF contamination of the food and foodstuffs has great importance [4]. The AF content of the food and foodstuffs is very strictly controlled in all countries of the world. In Turkey, although the legal upper limits in food for laying hens are 10 *μ*g/kg for AFB_1_ and 20 *μ*g/kg for AF [23], these limits are frequently neglected. So, the process of the detoxifying AF in contaminated food and foodstuffs has been coming into prominence. Although several methods have been examined, economic and practical methods for detoxifying AF-contaminated foodstuffs are very limited. The physical (heat inactivation, irradiation, solvent extraction, and use of adsorbents) and chemical (ammonia and ammonia-related compounds) methods have been administrated [4, 6]. Nonnutritive and inert adsorbents in the diet to bind AF and reduce the absorption of AF from the gastrointestinal tract has the new approach to the problem. Several studies have been performed using aluminosilicates [24], clinoptilolite [5, 14, 19], bentonites [25], phyllosilicates [26], polyvinyl polypyrrolidone (PVPP) [4], and activated charcoal [27] for detoxifying AF in contaminated food and foodstuffs.

In mammals, a few studies dealing with the prevention of dietary AF were performed. Lindemann et al. [28] have reported that the addition of hydrated sodium calcium aluminosilicate (HSCA) to the AF-contaminated diet restored the clinical chemistry profile in swine. Mayura et al. [29] have indicakted the effectiveness of HSCA in reducing the bioavailability of AFB_1_ and preventing the developmental toxicity of AF in rats. The same investigators [29] have pointed out the potential for significant hidden risks associated with the inclusion of nonselective AF binders in feed. So, they suggested that AF sorbents should be rigorously tested individually and thoroughly characterized in vivo, paying particular attention to their effectiveness and safety in sensitive animal models and their potential for deteriorated interactions.

In the present study, ANAE-positive peripheral blood lymphocyte counts were significantly affected by AF treatment. However, the addition of glucomannan to AF containing diet significantly recovered the adverse effects of AF. The values were numerically intermediate between controls and those of AF-given animals. It was observed that the restoration of the lymphoid tissue in rabbits fed with diet containing glucomannan combination with AF. These findings were well-adjusted with Çelik et al. [4] having investigated the ameliorative effects of PVPP on lymphoid tissue in chicks. *Saccharomyces cerevisiae* (SCE), another nontoxic adsorbent and its cell-wall component (mannan oligosaccharide), binds toxic molecules and protects their absorption from the gastrointestinal tract [5, 6]. Parlat et al. [5] reported that SCE provided significant improvement in aflatoxicosis cases in quail chicks. Basmacioglu et al. [6] have observed that the addition of esterified glucomannan to AF-containing diet significantly recovered the adverse effects of AF on performance, biochemical, and hematological values of broilers.

More importantly, in the present study, the dose level of AF was too low to cause clinical symptoms and serious histopathological lesions in lymphoid tissue. In fact, our findings have demonstrated that the dose level used in this study has caused slightly lymphoid cell depletion in lymphoid organs. However, the results obtained from ANAE histochemistry performed on peripheral blood lymphocyte were more dramatic than findings observed in the tissue sections. These results have suggested that the low level of AF could not cause severe tissue lesions, but produced significant decline in ANAE-positive peripheral blood lymphocyte levels. This situation makes AF contamination more serious problem in farms. Because low-level AF-containing food does not cause distinct clinical symptoms or pathological lesions, the toxication may be overlooked. Consequently, this toxication might trigger more serious trouble until the real problem is realized.

## Figures and Tables

**Table 1:** ANAE-positive PBL rates of control and experimental groups at the end of the study.

Treatment (*N* = 6)	ANAE (+) PBL percentages $\left(\overset{®}{X}\pm \text{SE}\right)$
Control	34.83 ± 1.04^a^
GM	32.33 ± 1.33^ab^
AF-GM	30.00 ± 1.23^b^
AF-treated	26.17 ± 0.65^c^

^a–c^Values within a column with no common superscripts are significantly different (*P* < 0.05).
